# Focus shifts in contextual and lexical cue interactions in GPT models

**DOI:** 10.1371/journal.pone.0351924

**Published:** 2026-06-18

**Authors:** Wonil Chung, Keonwoo Koo

**Affiliations:** Department of English Language and Literature, Dongguk University, Seoul, South Korea; Philadelphia University, JORDAN

## Abstract

Transformer-based language models have demonstrated sensitivity to a range of linguistic dependencies, yet it remains unclear how they represent information-structural focus and integrate discourse and lexical focus cues during ellipsis resolution. We investigated GPT-style models’ interpretation of elliptical remnant continuations in double-object constructions by manipulating contextual focus via preceding interrogatives (*who* vs. *what*) and lexical focus via the particle *only*, whose surface position was varied. Using word-by-word surprisal as an index of processing difficulty, we conducted three experiments with GPT-2 models (Small–XL) and GPT-Neo. In Experiment 1 (no *only*), models robustly tracked the *wh*-induced discourse focus, assigning higher surprisal to remnants that mismatched the contextually focused constituent. In Experiment 2 (*only* preceding the indirect object), contextual focus continued to dominate, indicating that discourse cues were maintained despite the presence of a competing lexical marker. In Experiment 3 (*only* preceding the direct object), lexical focus effects became stronger: models favored remnants aligned with the lexically biased direct object, consistent with locality-based cue weighting when *only* is adjacent to that object. Comparisons with human reaction-time data revealed broad convergence in contextual-focus sensitivity but divergence when the remnant was compatible with one cue but not the other, with GPT-style models exhibiting a stronger bias toward alignment with *only* than humans. Together, these findings suggest that the tested models maintain discourse-level focus representations while integrating multiple focus cues in a proximity-sensitive manner, revealing both overlap and limits in their alignment with human processing.

## 1. Introduction

Understanding language comprehension crucially involves the establishment and maintenance of information structure, particularly the assignment of *focus* within a sentence and across discourse [1,2]. *Focus* determines which part of an utterance contributes new, contrastive, or discourse-relevant information, and it guides interpretation by shaping expectations about upcoming material [3]. In natural language, *focus* can be introduced and modulated through multiple cues, including discourse context (e.g., interrogative questions), lexical operators such as *only*, and structural properties of sentences (e.g., *John wondered who Sally would pass the apples. Sally passed only the children the apples*). Successful comprehension, therefore, requires integrating these cues incrementally, especially when they converge or compete.

Interrogative contexts are a powerful discourse device for establishing focus, as they specify which constituent in a subsequent answer addresses the current question under discussion. Psycholinguistic research has shown that such contextually induced focus is maintained across sentence boundaries and influences online processing, with increased difficulty observed when incoming material fails to align with discourse expectations [4,2]. In addition to contextual cues, focus can also be lexically encoded by particles such as *only*, which introduce an exhaustive interpretation and bias focus toward a particular constituent within their syntactic domain. Importantly, contextual and lexical cues to focus may either align or diverge, raising the question of how focus is computed under cue competition during real-time processing.

These days, Neural language models (NLMs) generate linguistic output by estimating the probability of upcoming words given prior context. Through large-scale pre-training on human language data, these models can acquire linguistic representations that support context-sensitive predictions [5,6,7]. This predictive setup allows them to assign graded expectations to continuations, which provides a useful computational analogue for studying expectation-based aspects of sentence processing [8,9]. Surprisal, defined as the negative log-probability of a word given its context, has been widely adopted as a computational measure of processing difficulty, given its relationship with human reaction times [10,11,12]. This enables us to investigate how language models respond to linguistically relevant cues, including those related to information structure.

Despite growing interest in comparing human and NLM sentence processing[], it remains unclear to what extent transformer-based models represent discourse-level focus and how they integrate contextual and lexical focus cues when these cues bias different constituents. Previous human study [2], demonstrated that readers are sensitive to both interrogative context and the focus particle *only* when processing double-object constructions followed by elliptical remnants, and that the influence of *only* depends on its surface position. This paradigm, therefore, provides a test case for examining whether language models exhibit similar sensitivities or whether their predictions are driven primarily by local lexical cues.

In the present study, we investigate how transformer-based GPT models process information structure on double-object constructions, using interrogative contexts to establish discourse focus and the particle *only* to introduce lexical focus. Interrogative *wh*-questions are used as tools for manipulating discourse expectation rather than as objects of syntactic investigation. Specifically, we examine whether models maintain expectations established by discourse context, how these expectations interact with lexically encoded focus introduced by *only,* and whether model behavior varies depending on the linear placement of the lexical focus marker when interpreting elliptical remnant continuations.

To achieve our goal, we analyze GPT-2 models of different sizes (small, medium, large, XL) as well as GPT-Neo’s surprisal, a metric indicating the degree of unexpectedness in word-by-word processing [13,9]. Therefore, by examining surprisal patterns across critical sentence regions, we can evaluate whether models maintain discourse-level focus expectations, whether local lexical cues dominate, and how these tendencies compare to human processing profiles.

Based on this framework, the present study addresses the following research questions:

1. To what extent do GPT models maintain expectations established by discourse context in double-object constructions?
2. How do expectations derived from contextual questions and lexically encoded focus (*only*) jointly influence surprisal during the interpretations of elliptical remnants?
3. How does the linear position of the lexical focus marker affect model sensitivity to expectation congruency across different GPT-style models?

To investigate these questions, we employ three experiments in a psycholinguistic paradigm. The stimuli are adopted from [2], enabling direct comparisons with human results. By analyzing NLMs across experimental conditions, the study aims to clarify how transformer-based models process discourse-level information structure and to what extent predictive mechanisms align with or diverge from human sentence processing.

This paper is structured as follows: Chapter 2 reviews prior studies on focus identification in humans and related research involving NLMs. Chapter 3 presents our experiments examining the interaction of contextual focus and the lexical focus particle *only* in the GPT-2 series and GPT-Neo. Chapter 4 provides a general discussion of the findings. Finally, Chapter 5 concludes with insights into how GPT models process language regarding our experiments.

## 2. Background and related work

### 2.1. Previous studies on focus identification in humans

Researchers in linguistics have investigated the concept of *focus* to describe various interconnected phenomena. In these fields, focus generally refers to the element of a sentence or discourse that is emphasized or highlighted, drawing the listener’s or reader’s attention to specific information [14,15,16,17]. For example, *JOHN bought the apples*, the speaker highlights the identity of the buyer rather than the event itself (Note that in this paper, capital letters in examples indicate the stressed constituent). Focus, therefore, marks which part of the message the speaker treats as most relevant. In addition, linguists study *focus* to understand how speakers use intonation, word order, and other cues to signal importance, introduce new information, or create contrast with alternatives [18,14,19,20,21,4,2]. For instance, *Only Mary passed the exam* implies that alternative individuals did not pass, illustrating how focus evokes a set of contrasting possibilities. These approaches emphasize the critical role of focus in influencing how meaning is constructed, conveyed, and understood across diverse contexts. In discourse, listeners track this highlighted information across sentences: (e.g., A: *Who did John praise yesterday*? B: *He praised MARY, not Sue*). Here, the contrastive interpretation depends on maintaining attention to the previously introduced variable [21].

While the examples above illustrate the intuitive notion of highlighting, *focus* also has a systemic structural distribution within question-answer pairs. A question introduces a variable and thereby signals an anticipated focus in the answer, provided that the answer resolves that variable [19,22]. According to [23], there is a correlation between questions and the position of focus in answers. For instance, in question-answer pairs, (1c) is an appropriate answer to (1a) because the stressed phrase MARY corresponds to the variable introduced by the *wh*-question, whereas (1d) answers (1b). The question sets the focus of the answer by establishing a semantic framework that includes a range of possible responses, both true and false [16]. The focus in (1c) and (1d) represents informational focus, introducing new, non-presupposed information [24,25]. This type of focus is typical in question-answer pairs, such as those in (1a) and (1b). In English, focus is commonly realized through prosodic stress. In the following examples, capital letters indicate the stressed constituent.

(1) a: Who cut Bill down to size?
b: Who did Mary cut down to size?
c: **MARY** cut Bill down to size.
d: Mary cut **BILL** down to size.

Importantly, (1c) is not a felicitous answer to (1b), and (1d) is not a felicitous answer to (1a): each question introduces a different *wh*-variable, and only an answer that resolves that variable can serve as the informational focus of the corresponding question.

Experimental studies also have indicated that listeners understand information more easily when prosodic cues mark the focus, as opposed to when these cues are absent [26,27,28,29]. Also, it has shown that listeners more readily perceive focused over non-focused information [22,30] and that focused information is better retained in mind [31,32,33,34]. In addition, prosodic marking of focus has been found to influence interpretations of ambiguous sentences such as: *I asked the pretty little girl WHO is cold / who is COLD.* [33]. Furthermore, some studies have revealed that sentence focus structure is marked by focus-sensitive particles like *only* [20,4,2], and also by contextual cues [18,14,19,20,35,36,2].

[37] investigated how a preceding interrogative context affects focus processing by conducting an eye movement experiment. They analyzed how the *wh*-element in a question determines the location of focus in the corresponding answer. Using identical answer sentences, question-answer pairs were manipulated to determine which constituent was interpreted as new information. In (2), the *where*-question introduced a location variable, so focus fell on the locative phrase (e.g., *in the underground bunker*) within the answer sentence. In contrast, in (3), the *what*-question introduced an object variable, and focus shifted to the object (e.g., *cards*) within the answer sentence.

(2) a. Where were the soldiers?
b. The soldiers /**in the underground bunker** /were playing /cards /to relieve their boredom.

(3) a. What were the soldiers playing?
b. The soldiers /in the underground bunker /were playing /**cards** /to relieve their boredom.

Their findings showed that native speakers spent more time reading the focused portions of target sentences compared to the unfocused ones. [37] suggested that readers encode focused information more thoroughly and that the preceding context can influence focus identification.

Focus particles, such as *only,* associate with a focused element in a sentence to establish exhaustive focus, specifying a contrast between the referenced component and its alternatives [38,39,40–42,43]. According to [43], focus has a truth-conditional effect in the context of *only.* In an example from [44], *Mary introduced Bill and Tom to Sue*, with no other introductions occurring. In this context, (4a) is false and (4b) is true. Notably, the two sentences differ *only* in the location of prosodic stress, which marks the focus.

(4) a. Mary only introduced **BILL** to Sue.
b. Mary only introduced Bill to **SUE.**

Regarding focus distribution, the direct object *Bill* is focused in (4a), whereas the indirect object *Sue* is focused in (4b). (4a) implies that Bill alone was introduced to Sue, and (4b) implies that Bill was introduced exclusively to Sue and no one else [38]. [38] suggested that when prosody emphasizes the focused constituent within the particle’s scope, the particle associates with this constituent. However, during silent reading, explicit prosodic cues are absent. Thus, when processing written sentences, other factors will influence focus identification, especially the grammatical cues provided by the surface placement of the focus particle [45].

[4] investigated whether the placement of the particle *only* in different surface positions influenced focus identification during reading, using an eye tracking experiment. English natives were presented with double-object sentences like (5a-b), where the elliptical remnant in the second part (*her father/the pepper*) of a sentence is composed of a congruent contrast with either the indirect or direct object in the first part. (5a) is to assess the effect of locating *only* next to either the direct or the indirect object in double-object sentences, and (5b) is to examine the processing of sentences that did not include the focus particle *only*. Note that in all the sentences, in this experiment and the other experiments in this paper, the indirect object always precedes the direct object.

(5) a. Double-object sentences with *only*

At dinner, Jane passed [only] her mother [only] the salt but not (her father/the pepper) as well because she couldn’t reach.

b. Double-object sentence without *only*

At dinner, Jane passed her mother the salt but not (her father/the pepper) as well because she couldn’t reach.

In (5b), without the particle *only,* there were no significant reading-time effects between indirect objects (356 ms) and direct objects (419 ms) in the remnant region. However, in (5a), which included *only*, reading times for remnants were shorter in conditions where the indirect object was congruent (congruent: 745 ms vs. incongruent: 811 ms) when *only* preceded the indirect object, and shorter in conditions where the direct object was congruent (congruent: 763 ms vs. incongruent: 835 ms) when the particle *only* preceded the direct object. The congruency effect was significant in the remnant region. The congruency effect was weaker in the post-remnant region when the particle *only* was placed before the direct object (congruent: 489 ms vs. incongruent: 515 ms) compared to when it preceded the indirect object (congruent: 745 ms vs. incongruent: 811 ms). Significant reading-time differences indicated that the surface position of the focus particle influenced processing. [4] concluded that the position of a focus particle affects focus identification during real-time sentence comprehension, showing that the focus particle *only* was typically associated with the adjacent subsequent constituent.

Similar to the approach used by [4,2] investigated how contextual and lexical focus cues interact in double-object constructions, including interrogative contexts, using three eye-movement experiments while reading sentences such as (6). They used context to emphasize either the indirect or direct object in a double-object sentence, followed by a remnant continuation that presented either a congruous (6a: *who* … *the grownups*) or incongruous (6b: *what* … *the cherries*) contrast with the contextually focused object.

(6) a. IO-context (focus on the indirect object, e.g., *the children*)

John wondered who Sally would pass the apples. Sally passed [only] the children [only] the apples but not (the grownups/the cherries), because they did not want them.

b. DO-context (focus on the direct object, e.g., *the apples*)

John wondered what Sally would pass the children. Sally passed [only] the children [only] the apples but not (the cherries/the grownups), because they did not want them.

[2] investigated the effect of interrogative contexts on the processing of elliptical remnant constructions without the focus particle *only* in the first experiment. Their findings showed longer reading times for remnants that were incongruous with the contextually focused object, indicating that the context effectively specified focus. Furthermore, they revealed processing difficulties in the object region when focus was specified on the indirect object. That is, reading times of the *apples* were longer when the context included a *who* question than when it included a *what* question. This suggests that a preference for ‘given-before-new’ information influences reading times in the object’s region, indicating that the information-structural status of the objects influenced processing at an early stage.

In the second experiment, [2] investigated interrogative contexts together with the particle *only* before the indirect object (e.g., *John wondered* ***who/what*** *Sally would pass the apples. Sally passed* ***only*** *the children the apples but not* ***the grownups/cherries****, …*). Their eye-tracking results showed that the remnants were generally read faster when they contrasted with the constituent highlighted by the preceding wh-question, indicating robust sensitivity to discourse-induced focus. They also reported that the surface position of *only* can modulate processing, but that discourse focus remains a strong predictor of remnant processing difficulty in this configuration.

In their third experiment, [2] placed *only* between the indirect and direct objects (e.g., *John wondered* ***who/what*** *Sally would pass the apples. Sally passed the children* ***only*** *the apples but not* ***the cherries****/****grownups****, …*). They found a significant difference between the IO-context and DO-context due to processing difficulties in the IO-context within the object region. In addition, interaction between lexical and contextual focus cues was observed in the remnant region. Furthermore, they showed Congruency effects that were evident in the DO-context but not in the IO-context, because there was no difference in reaction time between *the grownups/the cherries* in (6a). Note that [2] reported the reaction times as follows: In (6a): at the region *the grownups* 885 ms; at the region *the cherries* 896 ms; In (6b): at the region *the cherries* 884 ms; at the region *the grownups* 1018 ms. They concluded that when focus cues specify focus on different constituents, there was no clear preference for remnants to align with either the contextually or lexically focused constituent, indicating that cue conflict was not resolved.

In our experiment investigating focus processing in transformer-based GPT models, we use experimental materials from [2], comparing human reading behavior with model predictions and investigating whether GPT models are sensitive to contextual focus cues or the position of lexical focus cues.

### 2.2. Previous work on NLMs

Transformer-based language models, introduced by [46], are grounded in the transformer architecture and use attention mechanisms to weigh the importance of each word in a sentence in relation to the others. This mechanism allows them to effectively capture dependencies and relationships between words across long contexts, surpassing the performance of previous architectures, such as RNNs and LSTM networks. Therefore, the GPT series is highly effective for linguistic research because of its capability to preserve contextual coherence and process language left-to-right, similar to humans [47,48,49,46].

GPT-style models are trained on large-scale text datasets using an auto-regressive language modeling approach, which enables them to predict the next word in a sequence based on prior context. For example, in a sentence such as *the cat is on the mat,* the model presents the predicted value of *on* in the sentence as p(on|the, cat, is) based on its conditional probabilities given the preceding words. This auto-regressive approach aligns well with tasks that involve understanding of sequential data, contributing to the models’ ability to generate fluent and contextually appropriate text.

To evaluate the language learning and processing performance of GPT-style models, recent studies have employed controlled psycholinguistic testing to assess grammatical knowledge [50,51,52,53,54,55,56,1,11,12,57]. Additionally, researchers have examined these models by analyzing the surprisal, which reflects the model’s expectations about linguistic input more generally. The following formula indicates the surprisal (negative log-probability), $S(w_{i})$ of a sentence’s *i*-th word.


$$\begin{array}{c} S\left(w_{i}\right)=-\text{log }p\left(w_{i}|c\right)=-\text{log}(p\left(w_{i}|w_{i}\ldots w_{i-\text{1}}\right)) \end{array}$$

In Surprisal Theory [5,9], surprisal has been shown to correlate with human sentence processing difficulty, so a word with a high surprisal is correlated with high reading times. Many language model studies have used surprisal as to assess their processing of syntactic information [1,11,12,58–64]. Especially, [11] reported that surprisal effectively predicts reading times across 11 languages in the mGPT model. In recent years, many researchers have investigated linguistic phenomena using GPT-based models [61,65,57,11,1].

Building on these, the present study investigates whether GPT models, especially the GPT-2 series and GPT-Neo, represent the interaction between *wh*-dependencies, which serve as discourse-level cues, and focus markers like *only*, which operate at the sentence level, during the processing of elliptical remnants in double-object constructions. We approach this question by analyzing the surprisal produced by these models. GPT-2 (small, medium, large, XL) and GPT-Neo, both advanced transformer-based language models [66], have significantly advanced the field of natural language processing. While they share fundamental architectural similarities, GPT-Neo, developed by EleutherAI to replicate GPT-3’s architecture, boasts a substantially larger size, with up to 2.7 billion parameters compared to GPT-2’s maximum of 1.5 billion parameters. This larger size enables GPT-Neo to generate more sophisticated linguistic knowledge.

## 3. Methods

This study investigates how GPT-style models process double-object constructions containing contextual and lexical focus cues, followed by elliptical remnant continuations. Adopting a psycholinguistic framework, we used experimental materials adopted from [2] and collected word-by-word surprisal from GPT models, including GPT-2 (small, medium, large, XL) and GPT-Neo-1.3B. The models are publicly available at: https://huggingface.co/openai-community/gpt2(-medium/-large/-xl), and Neo model is available at: https://huggingface.co/EleutherAI/gpt-neo-1.3B.

In line with standard practice in computational psycholinguistics, we interpret surprisal as a linking hypothesis to humans’ reaction time [13,9,10]. In addition, since the tested models are trained with a subword tokenizer (e.g., BPE), we adopt [10]’s word-level surprisal method, negative log probabilities of subword tokens corresponding to *w*_t_ were summed to calculate *S*(*w*_t_)= -log*P*(*w*_t_ | *w*_1_…*w*_t-1_) according to the chain rule of conditional probabilities. Regarding the truncation policies of experimental sentences, the processing loop treated every sentence as a distinct sequence, which was explicitly initiated with the Beginning-of-Sentence (BOS) token.

In Experiment 1, we manipulated interrogative contexts to emphasize either the indirect or the direct object, to investigate whether contextual focus affects the processing of elliptical remnants. In Experiment 2, the focus particle *only* was positioned before the indirect object, while in Experiment 3, it was placed directly before the direct object. These experiments examined how the alignment or misalignment between contextual focus and lexical focus influences the processing of elliptical remnant constructions.

### 3.1. Experiment 1

Experiment 1 investigates whether interrogative contexts affect the processing of elliptical remnant constructions. In this experiment, the focus particle was absent, leaving the focus structure unmarked at the sentence level. As a result, remnant elements are expected to align with the contextually focused object, which was the only constituent carrying discourse-level focus.

#### 3.1.1. Materials.

The experimental materials were constructed using a 2 × 2 factorial design with WhFocus (match vs. mismatch) and Type of Remnant (indirect object (IO) or direct object (DO)) at the remnant position as the key factors. Each of the four conditions included 32 items, resulting in a total of 128 sentences. At the discourse level, interrogative contexts were used to adjust expectations about focus structure by directing focus to either the indirect or direct object, depending on the *wh*-element. At the sentence level, the focus particle was absent. Each ditransitive sentence was followed by a remnant continuation that either matched or mismatched the focus projected by the *wh*-focus. The materials were adopted from [2] and were manipulated as minimal pairs for controlled comparison across conditions (see examples 7a-d).

(7) a. who-IO: *wh*-focus match, IO-remnant

John wondered **who** Sally would pass the apples. Sally passed the children the apples but not the grownups, because they did not want them.

b. what-IO: *wh*-focus mismatch, IO-remnant

John wondered **what** Sally would pass the children. Sally passed the children the apples but not the grownups, because they did not want them.

c. what-DO: *wh*-focus match, DO-remnant

John wondered **what** Sally would pass the children. Sally passed the children the apples but not the cherries, because they did not want them.

d. who-DO: *wh*-focus mismatch, DO-remnant

John wondered **who** Sally would pass the apples. Sally passed the children the apples but not the cherries, because they did not want them.

#### 3.1.2. Analysis.

We analyzed surprisal at the remnant region using linear mixed-effects models. Fixed effects included WhFocus, Type of Remnant, and their interaction (WhFocus × Remnant). Random effect was included Items. We used contrast coding for the categorical predictors: WhFocus (who: + 0.5, what: −0.5) and Remnant (IO: + 0.5, DO: −0.5). These analyses were conducted with the *lmer* function of the lme4 package [67] in R version 4.0.2.

#### 3.1.3. Results.

##### 3.1.3.1. The results of the object region in the answer sentence.

**Fig 1:**
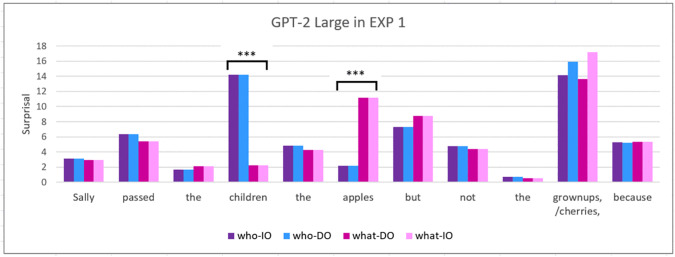
Mean Surprisal at each region in Experiment 1 within GPT2-LargeLarge.

Before analyzing the remnant region, we first examined surprisal patterns in the object regions as a function of *wh*-context (*who* vs *what*). Because remnant congruency is not yet determined at this stage, this analysis evaluates the effects of wh-context type rather than WhFocus. As illustrated in Fig 1, surprisal at the object region of the answer sentence varied significantly depending on the *wh*-element specified in the preceding interrogative context. When the interrogative context focused on the indirect object (*who*-context; e.g., *John wondered who Sally would pass the apples*), all models showed a significant effect of *wh*-context type (*p* < .001) at the indirect object region (e.g., *children*), with higher surprisal observed in conditions (7a, 7d) than in conditions (7b, 7c). In contrast, at the direct object region (e.g., *apples*) when the interrogative context focused on the direct object (*what*-context; e.g., *John wondered what Sally would pass the children*), all models also revealed a significant effect of *wh*-context type (*p* < .001), with higher surprisal observed in conditions (7b, 7c) than in conditions (7a, 7d).

##### 3.1.3.2. The results of the remnant region in the answer sentence.

**Fig 2:**
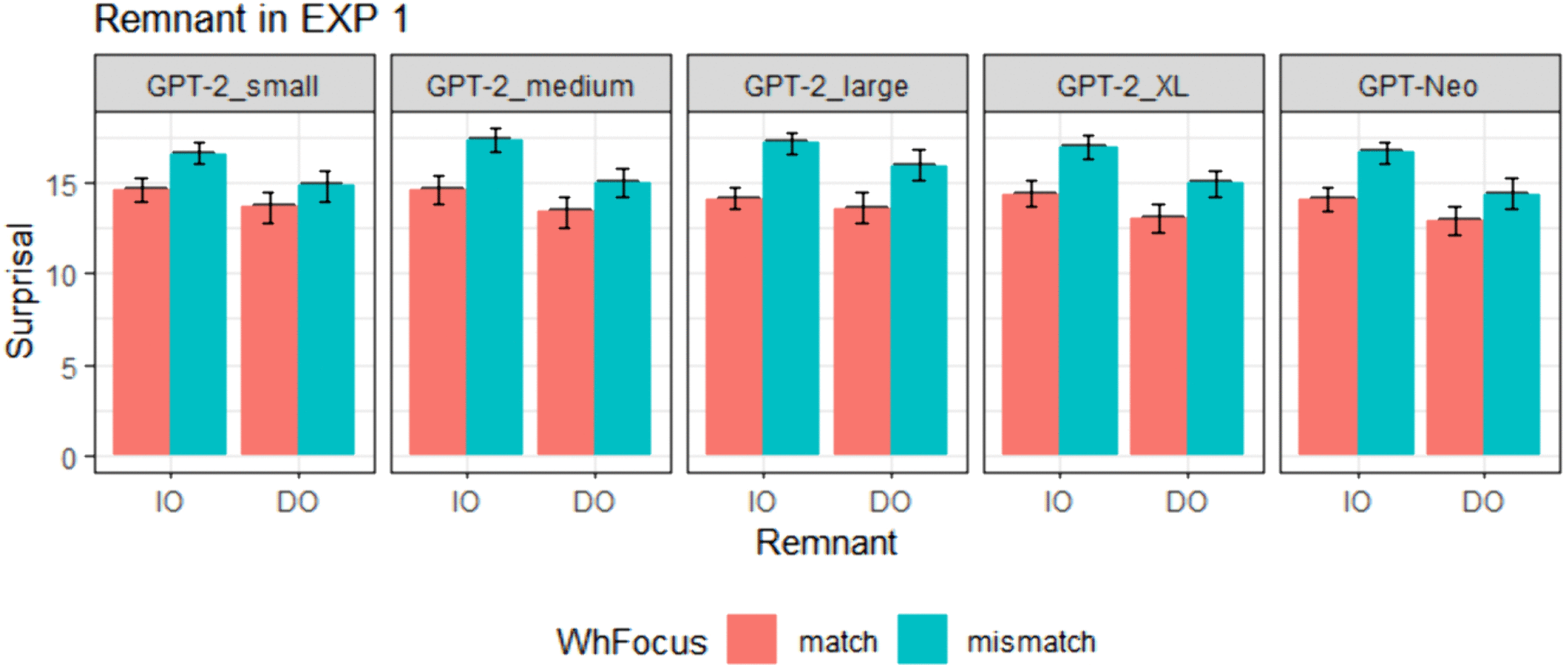
Sensitivity to *wh*-focus in the elliptical remnants in Experiment 1.

As shown in Fig 2, surprisal at the elliptical remnant region differed between match (7a, 7c) and mismatch (7b, 7d) conditions, reflecting the influence of the preceding interrogative context that established focus in the target sentence. In both IO-remnant and DO-remnant conditions, mismatch conditions yielded higher surprisal than match conditions, respectively. Fig 2 displays surprisal patterns across models, showing that the difference between match and mismatch conditions was more for IO-remnants than for DO-remnants.

To determine statistical significance in the remnant region, surprisal was analyzed using linear mixed-effects models, with WhFocus and Type of Remnant (ToR) treated as within-item factors across four conditions. As summarized in Table 1, all models showed a significant main effect of WhFocus (*p* < .01 or *p* < .001), driven by lower surprisal in the match conditions, which reflected the focus established by the prior interrogative context. Furthermore, a significant main effect of ToR (*p* < .05 or *p* < .01) was observed in all models except GPT2-Large, with the IO-Remnant generally yielding higher surprisal than the DO-Remnant across all levels of match. No significant interaction between WhFocus and ToR was observed.

**Table 1:** Fixed effects for the linear mixed-effect model predicting the surprisal in elliptical remnants in each model in Experiment 1.

	*GPT-2* *Small*		*GPT-2* *Medium*		*GPT-2* *Large*		*GPT-2* *XL*		*GPT-Neo*	
	b	t	b	t	b	t	B	t	b	t
Overall analysis										
(Intercept)	14.9	26.8***	15.1	28.2***	15.2	31.5***	14.9	32.9***	14.5	29.3***
WhFocus	−1.6	−2.7**	−2.2	−3.4***	−2.7	−4.5***	−2.3	−3.5***	−2.0	−3.4***
ToR	1.4	2.3*	1.8	2.7**	0.9	1.5	1.7	2.7**	1.7	2.9**
WhFocus* ToR	−0.8	−0.7	−1.3	−1.0	−0.8	−0.6	−0.7	−0.6	−1.0	−0.9
Pairwise analysis										
IO-Remnant	2.19*		2.85**		3.83***		2.76**		2.82**	
DO-Remnant	−0.97		−1.33		−1.97†		−1.78†		−1.37	

^a^Note: (1) Surprisal ~ WhFocus * ToR + (1 | item); (2) b: estimate; t: t-value

##### 3.1.3.3. Discussion.

Experiment 1 examined whether interrogative contexts affect the processing of elliptical remnant constructions in GPT-2 series and GPT-Neo. The results from the object region in the answer sentence exhibited higher surprisal for the focused portions of target sentences in question-answer pairs compared to the unfocused parts, indicating that the models were sensitive to *wh*-focus. This pattern suggests that tested models encode focus information established by discourse-level *wh*-questions (*who, what*), consistent with human findings in [37], that native speakers spent more time reading the focused portion of target sentences compared to the unfocused ones. Likewise, consistent with the human results reported in [2], GPT models revealed increased processing difficulty in each respective object region when that specific object was contextually focused. Specifically, as shown in Fig 1, surprisal at the indirect object (*children*) was higher when the context included a who-question (focus assigned to the indirect object; 7a and 7d) than when it included a *what*-question. Conversely, surprisal at the direct object (*apples*) was higher when the context included a *what*-question (focus assigned to the direct object; 7b and 7c) than when it included a who-question.

Furthermore, as in Fig 2, the lower surprisal in match conditions relative to mismatch conditions for both IO-remnant and DO-remnant conditions at elliptical remnants highlights those prior interrogative contexts effectively guided focus in subsequent target sentences, facilitating the processing of elliptical remnants.

Compared to human data reported in [2], all models showed a similar pattern: remnants that mismatched the contextually focused object yielded higher surprisal, paralleling longer reading times and increased processing difficulty observed in human comprehension.

### 3.2. Experiment 2

Experiment 2 investigated how alignment or misalignment between contextual focus and lexical focus influences the processing of elliptical remnants. As in Experiment 1, interrogative contexts were used to direct focus to either the indirect or the direct object, thereby establishing contextual focus. At the sentence level, lexical focus was manipulated through the inclusion of the particle *only,* which was positioned immediately before the indirect object. This configuration enabled contextual (WhFocus) and lexical (LexFocus) cues to assign focus to different constituents, thereby creating potentially mismatching predictions at the remnant position. Resolving such competition requires revising expectations, leading to increased processing cost. Accordingly, conditions in which contextual and lexical cues diverge are expected to result in higher surprisal than conditions where the two cues align.

At the remnant region, comprehenders retrieve the antecedent that is highlighted by the discourse context. If contextual focus guides interpretation, surprisal should increase whenever the remnant refers to a constituent that does not match the element focused by the *wh*-question, yielding a main effect of WhFocus without an interaction. The particle *only*, however, lexically assigns focus to the indirect object in all conditions. Consequently, cue mismatch arises only in the *what*-context, where the *wh-*question focuses the direct object while lexical focus targets the indirect object; in the *who*-context, both cues converge on the indirect object. If lexical focus is preferred, lexical focus would guide interpretation more strongly, producing an interaction between WhFocus and LexFocus, with mismatch effects appearing mainly in the *what*-context as in (8).

#### 3.2.1. Materials.

Each of the four conditions included 32 items. As in Experiment 1, interrogative contexts were used to adjust expectations about the upcoming focus structure by shifting contextual focus onto either the indirect or direct object. At the sentence level, lexical focus was manipulated by positioning *only* before the indirect object (see examples 8a-d).

The materials followed a 2 × 2 factorial design, WhFocus (match vs. mismatch) and LexFocus (match vs. mismatch). This design follows the [2], allowing the experiment to test how contextual and lexical focus cues interact at the point of remnant interpretation. This manipulation creates potential processing difficulty when contextual and lexical cues diverge. In *who*-contexts, congruous remnants (who-IO, 8a) align with both the contextually and lexically focused constituent, whereas incongruous remnants (who-DO, 8d) do not align with contextually focused constituents but match with the lexically focused constituent. In the *what*-context, congruous remnants (what-DO, 8c) align with the contextually focused constituent but not with the lexically focused constituent, while incongruous remnants (what-IO, 8b) align with the lexically focused constituent but not with the contextually focused constituent.

(8) a. who-IO: *wh*-focus match, lexical focus match

John wondered **who** Sally would pass the apples. Sally passed only the children the apples but not the **grownups**, because they did not want them.

b. what-IO: *wh*-focus mismatch, lexical focus match

John wondered **what** Sally would pass the children. Sally passed only the children the apples but not the **grownups**, because they did not want them.

c. what-DO: *wh*-focus match, lexical focus mismatch

John wondered **what** Sally would pass the children. Sally passed only the children the apples but not the **cherries**, because they did not want them.

d. who-DO: *wh*-focus mismatch, lexical focus mismatch

John wondered **who** Sally would pass the apples. Sally passed only the children the apples but not the **cherries**, because they did not want them.

#### 3.2.2. Analysis.

We analyzed surprisal at the remnant region using linear mixed-effects models. Fixed effects included WhFocus, LexFocus, and their interaction (WhFocus × LexFocus). Random effect was included Items. We used contrast coding for the categorical predictors: WhFocus (who: + 0.5, what: −0.5) and LexFocus (IO: + 0.5, DO: −0.5). These analyses were conducted with the *lmer* function of the lme4 package [67] in R version 4.0.2.

#### 3.2.3. Results.

##### 3.2.3.1 The results of the object region.

**Fig 3:**
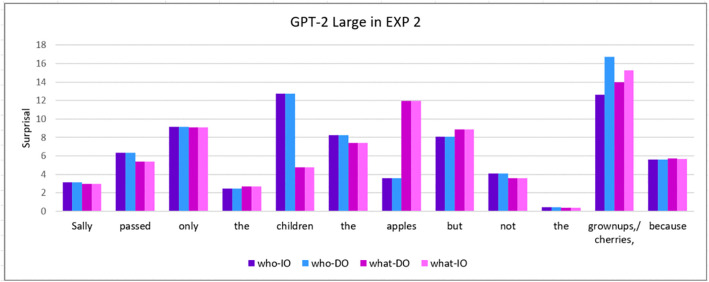
Mean Surprisal for all regions in Experiment 2 within GPT2-Large.

Before analyzing the remnant region, we first examined surprisal patterns in the object regions as a function of *wh*-context (*who* vs *what*). Because remnant congruency is not yet determined at this stage, this analysis evaluates the effects of wh-context type rather than WhFocus. As shown in Fig 3, similar to Experiment 1, surprisal at the object region revealed significant differences depending on the *wh*-context established by the interrogative context. At the indirect object region (e.g., *children*), a significant effect of *wh*-context type was found (*ps* < 0.001 across all models). Likewise, at the direct object region (e.g., *apples*), a significant effect of *wh*-context type (*ps* < 0.001 in all models) was observed, which means that the model was easy to process when the *wh*-phrase was *what* rather than *who* (see Fig 3).

##### 3.2.3.2. The results of the remnant region.

**Fig 4:**
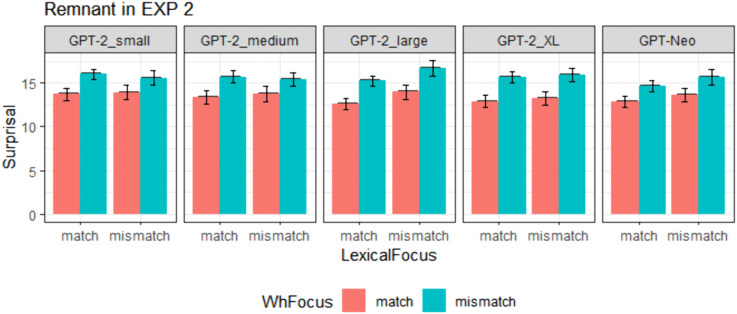
Mean Surprisal in the elliptical remnant region within each model.

Surprisal in the elliptical remnant region varied as a function of contextual congruency, with higher surprisal in mismatch than match conditions in both the *who*- and *what*-contexts (Fig 4). Across models, this pattern was consistent, indicating that models were primarily sensitive to the discourse focus established by the *wh*-question rather than to alignment between contextual and lexical focus cues.

For the statistical analyses, linear mixed-effects models were fitted to the surprisal, with WhFocus and LexFocus entered as within-item factors across four conditions. All models showed a significant main effect of WhFocus (*ps* < .01 or *p* < .001), indicating that surprisal was modulated by the focus specified in the preceding interrogative context (see Table 2). A significant main effect of LexFocus (*p* < .05) was additionally observed in GPT-2 Large. No significant interaction between WhFocus and LexFocus emerged in any model. Pairwise comparisons within the *who*-context showed a significant mismatch effect across the models (*ps* < .05 or <.01). In the *what*-context, the mismatch effect reached significance in GPT-2 Large and GPT-2 XL (*p* < .05).

**Table 2:** Fixed effects for the linear mixed-effect model predicting the surprisal in elliptical remnants in each model in Experiment 2.

	*GPT-2* *Small*		*GPT-2 Medium*		*GPT-2* *Large*		*GPT-2* *XL*		*GPT-Neo*	
	b	t	b	t	b	t	b	T	b	t
Overall analysis										
(Intercept)	14.82	27.33***	14.57	27.01***	14.65	29.03***	14.43	31.13***	14.19	27.43***
WhFocus	−1.98	−3.47***	−1.99	−3.08**	−2.75	−4.13***	−2.72	−4.21**	−1.94	−3.10**
LexFocus	0.08	0.14	−0.03	−0.06	−1.41	−2.18*	−0.26	−0.41	−0.85	−1.37
WhFocus* LexFocus	−0.64	−0.56	−0.67	−0.52	0.14	0.11	−0.12	−0.10	0.17	0.14
Pairwise analysis										
*who*-context	2.58*		2.30*		2.95**		2.92**		2.02*	
*what*-context	−1.42		−1.40		−2.25*		−2.42*		−1.71†	

^a^Note: (1) Surprisal ~ WhFocus * LexFocus + (1 | item); (2) b: estimate; t: t-value

#### 3.2.4. Discussion.

The results of Experiment 2 show that surprisal at the elliptical remnant was primarily modulated by the focus established in the preceding interrogative context. Consistent with human findings reported in [2], WhFocus match conditions yielded lower surprisal than WhFocus mismatch conditions. The significant main effect of WhFocus across models indicates that contextual focus continued to guide interpretation even when a lexical focus cue was introduced. Thus, placing *only* before the indirect object did not override the expectation generated by the *wh*-question, suggesting that contextual focus exerts a dominant influence on remnant resolution.

In contrast, the influence of LexFocus was limited, emerging only in GPT2-Large. This restricted effect suggests that lexical focus marking was not uniformly integrated across models and that sensitivity to *only* may depend on representational capacity. The absence of an interaction between WhFocus and LexFocus further indicates that contextual and lexical cues contributed independently rather than jointly shaping the surprisal pattern.

Taken together, the findings from Experiment 2 suggest that contextual focus remains the primary determinant of remnant interpretation, even in the presence of a competing lexical focus cue. However, because *only* fixed in the indirect-object position, lexical focus could not be fully crossed with contextual focus. Experiment 3 addresses this limitation by placing *only* before the direct object, enabling a complementary test of whether lexical focus influences remnant processing when the marked constituent differs from that specified by the context.

### 3.3. Experiment 3

Experiment 3 investigated how interrogative contexts influence the processing of elliptical remnant constructions when the focus particle *only* is placed before the direct object. The two factors, WhFocus and LexFocus, were defined by whether contextual focus (*who*-context vs *what*-context) and lexical focus (introduced by *only*) matched the remnant. Unlike Experiment 2, placing only before the direct object reversed the cue-alignment pattern. In the *who*-contexts (9a, 9d), the *wh*-question focuses on the indirect object, whereas *only* focuses on the direct object, creating a mismatched prediction. In the *what*-contexts (9b, 9c), both cues align on the direct object.

At the remnant, the processor must retrieve an antecedent compatible with the ellipsis. If interpretation is guided primarily by contextual focus, surprisal should depend on whether the remnant matches the constituent highlighted by the *wh-*question: remnants inconsistent with the contextual focus should yield higher surprisal, producing a main effect of WhFocus. Alternatively, if the element associated with *only* is prioritized, the remnants referring to the direct object should be preferred regardless of the question. Because the contextual and lexical cues mismatch in the who-contexts (who-IO vs. who-DO), in this case, lexical focus would guide interpretation strong preference to associate the particle with a subsequent element will lead to a preference for remnants that match the lexically focused constituent, that is, the direct object. In addition, this would produce an interaction between WhFocus and LexFocus or a robust LexFocus effect at the remnant.

#### 3.3.1. Materials.

Each of the four conditions included 32 items. As in Experiments 1 and 2, interrogative contexts established expectations about the upcoming focus structure by directing contextual focus to either the indirect or direct object. At the sentence level, lexical focus was manipulated by positioning *only* before the direct object (see examples 9a-d).

The materials followed a 2 × 2 factorial design, crossing WhFocus (match vs. mismatch) and LexFocus (match vs. mismatch), following the logic of [2]. In this configuration, cue alignment differs across contexts. In *what*-contexts, congruous remnants (what-DO, 9c) align with both contextual and lexical focus, whereas incongruous remnants (what-IO, 9b) align with neither. In the *who*-context, congruous remnants (who-IO, 9a) align with contextual focus but not lexical focus, while incongruous remnants (who-DO, 9d) align with the lexical focus but not contextual focus.

(9) a. who-IO: *wh*-focus match, lexical focus mismatch

John wondered **who** Sally would pass the apples. Sally passed the children only the apples but not the **grownups**, because they did not want them.

b. what-IO: *wh*- focus mismatch, lexical focus mismatch

John wondered **what** Sally would pass the children. Sally passed the children only the apples but not the **grownups**, because they did not want them.

c. what-DO: *wh*- focus match, lexical focus match

John wondered **what** Sally would pass the children. Sally passed the children only the apples but not the **cherries**, because they did not want them.

d. who-DO: *wh*- focus mismatch, lexical focus match

John wondered **who** Sally would pass the apples. Sally passed the children only the apples but not the **cherries**, because they did not want them.

#### 3.3.2. Analysis.

We analyzed surprisal at the remnant region using linear mixed-effects models. Fixed effects included WhFocus, LexFocus, and their interaction (WhFocus × LexFocus). Random effect was included Items. We used contrast coding for the categorical predictors: WhFocus (who: + 0.5, what: −0.5) and LexFocus (IO: + 0.5, DO: −0.5). These analyses were conducted with the *lmer* function of the lme4 package [67] in R version 4.0.2.

#### 3.3.3. Results

##### 3.3.3.1. The results of the object region.

**Fig 5:**
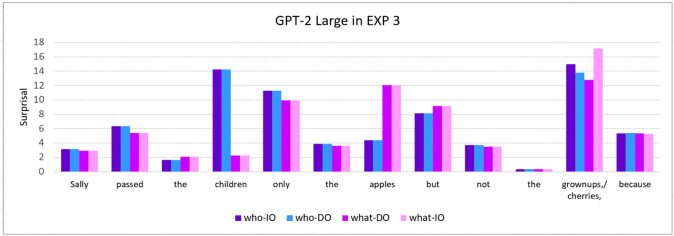
Mean Surprisal at each region in Experiment 3 within GPT2-Large.

Before analyzing the remnant region, we first examined surprisal patterns in the object regions as a function of *wh*-context (*who* vs *what*). Because remnant congruency is not yet determined at this stage, this analysis evaluates the effects of *wh*-context type rather than WhFocus. As illustrated in Fig 5, and consistent with Experiments 1 and 2, surprisal differed as a function of the *wh*-context established by the preceding interrogative sentence. At the indirect object region (e.g., *children*), a significant effect of *wh*-context type was observed across all models (*p*s < .001). A comparable pattern emerged at the direct object region (e.g., *apples*), where *wh*-context type also yielded a significant effect in all models (*p*s < .001).

##### 3.3.3.2. The results of the remnant region.

**Fig 6:**
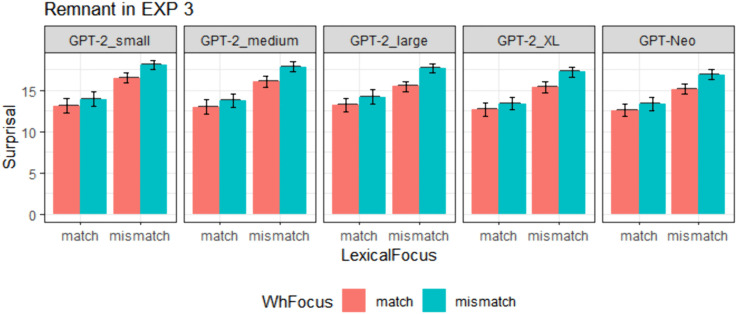
Comparing match and mismatch conditions at the remnant region.

Fig 6 below shows surprisal at the elliptical remnant across five language models (GPT-2 small, medium, large, XL, and GPT-neo), comparing match and mismatch conditions under WhFocus. Across all models, surprisal was higher in the lexical focus mismatch condition than in the lexical focus match condition, indicating increased processing cost when the remnant did not match the constituent associated with *only*.

Unlike Experiment 2, in which *only* was placed before the indirect object, Experiment 3, with *only* positioned before the direct object, showed a higher surprisal for mismatch conditions than match conditions in LexFocus compared to Experiment 2. In this configuration, surprisal differences were more strongly influenced by LexFocus than in Experiment 2. Specifically, conditions (9d) with *wh*-focus mismatch but lexical focus match showed lower surprisal than conditions (9a) with *wh*-focus match but lexical focus mismatch, suggesting that aligning with the lexically focused direct object reduced processing cost even when contextual expectations were violated.

For the statistical analyses, linear mixed-effects models were fitted to the surprisal, with WhFocus and LexFocus entered as within-item factors across four conditions. All models showed a significant main effect of WhFocus (*ps* < .05 or *p* < .01), indicating that surprisal was modulated by the focus specified in the preceding interrogative context (see Table 3). A significant main effect of LexFocus (*p*s < .001) was also observed, and the effect was larger than WhFocus, suggesting a strong contribution of lexical focus when *only* was positioned before the direct object. No interaction between WhFocus and LexFocus emerged in any model. Pairwise comparisons within the *who*-context showed a significant mismatch effect in GPT-2 Large, GPT-2 XL, and GPT-neo (*ps* < .05 or <.01), whereas no mismatch effect was observed in the *what*-context for any model.

**Table 3:** Fixed effects for the linear mixed-effect model predicting the surprisal in elliptical remnants in each model in Experiment 3.

	*GPT-2* *Small*		*GPT-2 Medium*		*GPT-2* *Large*		*GPT-2* *XL*		*GPT-Neo*	
	b	t	b	t	b	t	b	t	b	T
Overall analysis										
(Intercept)	15.39	29.81***	15.14	27.91***	15.12	30.86***	14.65	33.17***	14.47	30.39***
WhFocus	−1.22	−2.06*	−1.27	−2.01*	−1.62	−2.64**	−1.30	−2.06*	−1.27	−2.12*
LexFocus	−3.75	−6.34***	−3.58	−5.68***	−2.88	−4.68***	−3.29	−5.20***	−3.10	−5.20***
WhFocus* LexFocus	0.76	0.65	1.03	0.82	1.19	0.97	1.17	0.93	1.00	0.84
Pairwise analysis										
*who*-context	1.92†		1.87†		2.67**		2.13*		2.02*	
*what*-context	−0.70		−0.62		−0.87		−0.66		−0.70	

^a^Note: (1) Surprisal ~ WhFocus * LexFocus + (1 | item); (2) b: estimate; t: t-value

#### 3.3.4. Discussion.

The results of Experiment 3 show that surprisal at the elliptical remnant was influenced by both contextual and lexical focus cues when *only* was positioned before the direct object. Consistent with human findings from [2], lexical-focus match conditions yielded lower than lexical-focus mismatch conditions.

As in the previous experiments, a significant main effect of WhFocus indicates that models continued to track the focus established by the interrogative context, yielding higher surprisal when the remnant did not match the contextually expected constituent. This finding confirms that contextual focus remains a reliable cue guiding remnant interpretation, even when the lexical marking targets a different argument.

In contrast to Experiment 2, LexFocus exerted a stronger influence when *only* modified the direct object. The significant main effect of LexFocus across models suggests that lexical focus was more successfully integrated in this configuration. Pairwise comparisons further showed that mismatch effects emerged only in the *who-context*, and only in larger models (GPT-2 Large, GPT-2 XL, and GPT-Neo). Notably, conditions involving WhFocus mismatch, but LexFocus match were associated with lower surprisal than conditions involving WhFocus match but LexFocus mismatch, indicating that alignment with the lexically focused direct object reduced processing cost even when contextual expectations were violated. This pattern reflects a mismatch between two cues to antecedent retrieval: the discourse-level expectation established by the *wh*-question and the structural focus assigned by *only*. When these cues competed in the *wh*-context, alignment with the lexically focused direct object reduced surprisal, showing that lexical focus can mitigate this contextual-lexical cue mismatch when it highlights the more expected direct object.

Taken together, the findings from Experiment 3 suggest that the integration of contextual and lexical focus cues is sensitive to the position of the focus particle. Whereas lexical focus marking on the indirect object had limited impact in Experiment 2, modifying the direct object strengthened the influence of *only*, particularly in larger models capable of maintaining multiple cues. These results point to a graded sensitivity to cue competition and indicate that lexical focus may be weighted more heavily when applied to the direct object. Experiment 3, therefore, complements the previous findings by demonstrating that contextual focus remains dominant, but lexical focus can meaningfully modulate remnant interpretation under specific structural conditions, providing a foundation for the broader conclusions developed in the General Discussion.

##### 3.3.4.1. Comparison between human and GPT models.

**Fig 7:**
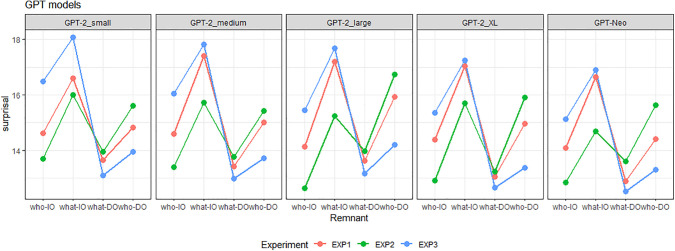
Surprisal across four remnant types in Experiments 1-3 for five model.

**Fig 8:**
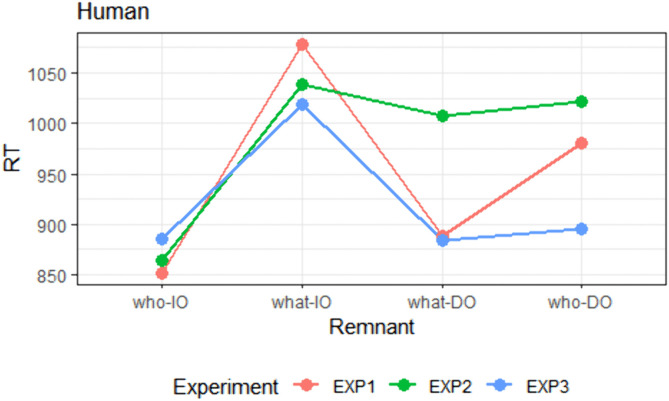
Mean reaction times across four remnant types in Experiment 1-3. *Adopted from Sauermann et al. (2013)*.

To assess whether GPT-style models parallel human sensitivity to focus-related cues during ellipsis resolution, we compared five GPT-style models’ behavior patterns with humans’ results from [2] across the three experiments (see also Fig 7 and 8). This comparison allows us to evaluate whether both systems exhibit similar condition rankings and whether the manipulation of contextual and lexical focus produces comparable processing consequences.

As shown in Fig 7, across all models, a highly consistent pattern emerged, which displayed the same qualitative response to the available focus cues. In Experiment 2, where *only* was positioned before the indirect object, WhFocus dominated: who-IO and what-DO conditions yielded lower surprisal than the mismatch conditions, indicating that the test models reliably encoded the *wh-*focus established by the interrogative context and incurred a processing penalty when the remnant violated those expectations. In Experiment 3, where *only* was positioned before the direct object, LexFocus exerted a stronger influence: what-DO and who-DO conditions showed lower surprisal than the lexical mismatch conditions. Thus, rather than responding equally to both cues, the models shifted sensitivity depending on which cue provided the stronger structural signal, with larger models (GPT2-Large, GPT2-XL, and GPT-Neo) showing the clearest effects.

The models exhibited a pattern broadly consistent with the human findings in [2], showing higher reaction times for mismatch than match conditions in both *who*-contexts and *what*-contexts. Relative to the human data, all models displayed a similar pattern in Experiments 1 and 2, with higher surprisal in mismatch than match conditions (see Fig 7 and Fig 8). Experiment 3 revealed a partial divergence between human and model behavior. While both showed maximal difficulty in the double-mismatch condition, humans exhibited similar processing costs across the remaining conditions (what-DO: 884 ms; who-IO: 885 ms; who-DO: 896 ms; what-IO: 1018 ms, [2]), whereas models differentiated them according to lexical-focus alignment. Specifically, the models showed the who-DO condition, which involved *wh*-focus mismatch but lexical-focus match, yielded lower surprisal than the who-IO condition, despite the presence of mismatching contextual and lexical cues. This pattern is consistent with the locality bias reported in [4], whereby the focus particle *only* tends to associate with the immediately following constituent. Accordingly, when contextual focus and lexical focus diverged, the tested models prioritized alignment with the lexically focused direct object.

## 4. General discussion

Across three experiments, the present study examined whether transformer-based language models are sensitive to contextual and lexical focus cues during the interpretation of elliptical remnant constructions. The findings from **Experiment 1** demonstrated that the tested models reliably encoded the *wh*-dependency established by interrogative contexts, showing higher surprisal when the remnant did not match the contextually focused constituent. This pattern indicates that discourse-level focus information is maintained across sentence boundaries, consistent with evidence that focused elements receive enhanced processing in human comprehension [37]. The results align with recent surprisal-based work showing that transformer models capture hierarchical dependencies beyond surface statistics [1]. Comparable effects reported in [2] suggest that GPT models and humans both experience processing difficulty when remnant interpretation mismatches with the prior discourse focus.

**Experiment 2** introduced lexical focus marking through *only* positioned before the indirect object to test whether local cueing could override contextual focus. The significant main effect of WhFocus across models indicated that contextual focus remained the dominant factor shaping surprisal, while LexFocus effects emerged only in GPT2-Large. The absence of an interaction between WhFocus and LexFocus supports the interpretation that contextual and lexical cues contributed independently rather than jointly influencing remnant resolution. From a retrieval perspective, these results suggest that models successfully maintained the *wh*-focus across intervening material, consistent with cue-based memory retrieval accounts in which stored features guide interpretation at the retrieval sites [7,68–71]. Because *only* was fixed in the indirect object position, its contribution did not fully compete with contextual expectations.

In contrast, **Experiment 3** revealed a different pattern when *only* positioned before the direct object. Although contextual focus continued to exert a robust influence, LexFocus produced a large effect across models, and larger models (GPT2-Large, GPT2-XL, GPT-Neo) showed reduced surprisal when the remnant aligned with the lexically focused direct object, even when the *wh*-question was violated. Importantly, the linear distance between Experiment 2 and 3 is minimal: *only* is separated from the remnant by just one additional noun phrase, so the effect cannot be attributed to a large linear distance change. Rather, the results suggest sensitivity to locality: when the focused constituent is immediately adjacent to the retrieval site, lexical focus exerts greater influence. This interpretation is consistent with evidence that retrieval and cue weighting are highly sensitive even to small recency differences [72,73,17,32,4] and with proposals that transformer attention assigns greater weight to recent or local cues [74,75]. Together, these findings show that the tested models encode contextual focus in a human-like manner, maintain *wh*-focus information across sentences, and incur processing cost in cue mismatch conditions. However, the divergence observed in Experiment 3 indicates that models differ from humans in how competing cues are integrated. In the tested models, both WhFocus and LexFocus exerted a strong influence, whereas human readers showed LexFocus. These results suggest that transformer models share some mechanisms with human processing, maintaining discourse-level information while responding to local cue strength, but do not fully replicate human cue weighting when contextual and lexical cues mismatch.

## 5. Conclusion

Across three experiments, this study showed that the tested models are sensitive to contextual focus during the interpretation of elliptical remnants, displaying higher surprisal when the remnant violated the *wh*-focus established by the interrogative context. These results indicate that the models maintain discourse-level focus across sentence boundaries and incur processing cost in cue mismatch conditions, broadly paralleling human patterns reported in previous work.

At the same time, the influence of lexical focus depended on structural position: *only* had a minimal impact when marking the indirect object but modulated surprisal when applied to the direct object, particularly in larger models. We interpret this asymmetry primarily as a locality effect, with argument prominence strengthening that bias. The direct object is easier to retrieve because it is linear to the remnant and corresponds to the verb’s central argument (the transferred item), making it a more accessible antecedent.

Overall, the tested GPT-style models encode contextual focus in a human-like manner but diverge from humans when contextual and lexical cues compete, suggesting a stronger reliance on locality-based retrieval heuristics.
